# Toward quantitative neuroimaging biomarkers for Friedreich's ataxia at 7 Tesla: Susceptibility mapping, diffusion imaging, *R*_2_ and *R*_1_ relaxometry

**DOI:** 10.1002/jnr.24701

**Published:** 2020-07-30

**Authors:** Sina Straub, Stephanie Mangesius, Julian Emmerich, Elisabetta Indelicato, Wolfgang Nachbauer, Katja S. Degenhardt, Mark E. Ladd, Sylvia Boesch, Elke R. Gizewski

**Affiliations:** ^1^ Division of Medical Physics German Cancer Research Center (DKFZ) Heidelberg Germany; ^2^ Department of Neuroradiology Medical University of Innsbruck Innsbruck Austria; ^3^ Neuroimaging Core Facility Medical University of Innsbruck Innsbruck Austria; ^4^ Faculty of Physics and Astronomy Heidelberg University Heidelberg Germany; ^5^ Department of Neurology Medical University of Innsbruck Innsbruck Austria; ^6^ Faculty of Medicine Heidelberg University Heidelberg Germany

**Keywords:** brainstem, cerebellum, diffusion imaging, fiber tracts, Friedreich's ataxia, magnetic resonance imaging, midbrain, MR relaxometry, nuclei, quantitative MRI, quantitative susceptibility mapping

## Abstract

Friedreich's ataxia (FRDA) is a rare genetic disorder leading to degenerative processes. So far, no effective treatment has been found. Therefore, it is important to assist the development of medication with imaging biomarkers reflecting disease status and progress. Ten FRDA patients (mean age 37 ± 14 years; four female) and 10 age‐ and sex‐matched controls were included. Acquisition of magnetic resonance imaging (MRI) data for quantitative susceptibility mapping, *R*_1_, *R*_2_ relaxometry and diffusion imaging was performed at 7 Tesla. Results of volume of interest (VOI)‐based analyses of the quantitative data were compared with a voxel‐based morphometry (VBM) evaluation. Differences between patients and controls were assessed using the analysis of covariance (ANCOVA; *p* < 0.01) with age and sex as covariates, effect size of group differences, and correlations with disease characteristics with Spearman correlation coefficient. For the VBM analysis, a statistical threshold of 0.001 for uncorrected and 0.05 for corrected *p*‐values was used. Statistically significant differences between FRDA patients and controls were found in five out of twelve investigated structures, and statistically significant correlations with disease characteristics were revealed. Moreover, VBM revealed significant white matter atrophy within regions of the brainstem, and the cerebellum. These regions overlapped partially with brain regions for which significant differences between healthy controls and patients were found in the VOI‐based quantitative MRI evaluation. It was shown that two independent analyses provided overlapping results. Moreover, positive results on correlations with disease characteristics were found, indicating that these quantitative MRI parameters could provide more detailed information and assist the search for effective treatments.

## Graphical abstract

Quantitative 7 Tesla MRI (susceptibility, relaxation, fractional anisotropy) found differences between ten Friedreich's ataxia patients and matched healthy controls in eight structures of brainstem and cerebellum which correlated with disease characteristics. Voxel‐based morphometry revealed white matter atrophy in partially overlapping volumes of interest (VOI) used for the VOI‐based evaluation. *Statistically significant differences between patients and controls; middle cerebellar peduncle (MCP), pontine crossing tract (PCT), corticospinal tract (CS), medial lemniscus (ML), inferior cerebellar peduncle (ICP), superior cerebellar peduncle (SCP), cerebral peduncle (CP), posterior thalamic radiation (PTR), sagittal stratum (SS), dentate nuclei (DN), red nuclei (RN), substantia nigra (SN).

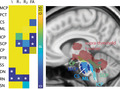

**Abbreviations**
ASPIRE: computationally efficient combination of multi‐channel phase data from multi‐echo acquisitions
*B*_1_: transverse RF magnetic field
BET: brain extraction tool
CP: cerebral peduncle
CS: corticospinal fibers
DARTEL: diffeomorphic anatomical registration using exponentiated lie algebra
DN: dentate nuclei
DTI: diffusion tensor imaging
EPI: echo planar imaging
FA: fractional anisotropy
FAST: FMRIB's automated segmentation tool
FLIRT: FMRIB's linear image registration tool
FMRIB: Oxford Centre for functional MRI of the brain
FNIRT: FMRIB's non‐linear image registration tool
FSL: FMRIB software library
GRAPPA: generalized autocalibrating partially parallel acquisitions
GRE: gradient echo
ICE: image calculation environment
ICP: inferior cerebellar peduncle
ID: identification number
JHU: Johns Hopkins University
MCP: middle cerebellar peduncle
MITK: Medical Imaging Interaction Toolkit
ML: medial lemniscus
MNI: Montreal Neurological Institute
MP2RAGE: magnetization prepared 2 rapid acquisition gradient echoes
MRI: magnetic resonance imaging
PCT: pontine crossing tract
PTR: posterior thalamic radiation (including optic radiation)
QSM: quantitative susceptibility mapping
RESOLVE: readout segmentation of long variable echo‐trains
RN: red nuclei
SCP: superior cerebellar peduncle
SN: substantia nigra
SNR: signal‐to‐noise ratio
SPM: statistical parametric mapping
SS: sagittal stratum (including inferior longitudinal fasciculus and inferior fronto‐occipital fasciculus)
STAR‐QSM: dipole inversion the streaking artifact reduction for QSM
STEAM: stimulated echo acquisition mode
TSE: turbo spin echo
VOI/VOIs: volume of interest/volumes of interest
V‐SHARP: variable‐kernel sophisticated harmonic artifact reduction for phase data

**Significance**

In five out of twelve investigated structures of the brainstem and the cerebellum, statistically significant differences were found in Friedreich's ataxia patients versus controls with quantitative magnetic resonance imaging at 7 Tesla (quantitative susceptibility mapping, *R*_1_, *R*_2_ relaxometry and diffusion imaging), and statistically significant correlations with disease characteristics were revealed. The regions in which voxel‐based morphometry showed significant white matter atrophy partially overlapped with the brain regions for which significant differences between controls and patients were found in the volume‐of‐interest‐based quantitative MRI evaluation. Quantitative MRI parameters could provide more detailed information about the disease and assist the search for effective treatments.

## 1 INTRODUCTION

Friedreich's ataxia (FRDA) is a rare genetic disorder affecting the FXN gene which produces the protein frataxin within the mitochondria of the cells (Koeppen, 2011). More precisely, most patients inherit trinucleotide (GAA) expansions within the first intron of the FXN gene, and those with a higher number of repeats (>500) are more likely to present at an earlier age and with different clinical symptoms (Schols et al., 1997), whereas longer GAA repeats lead to lower frataxin levels (Sacca et al., 2011). A lack of frataxin disrupts the iron metabolism (Gibson, Koonin, Musco, Pastore, & Bork, 1996; Zhang et al., 2006) leading to degenerative processes especially in tissues with a high metabolic rate such as the heart, pancreas, as well as the nervous system (Koeppen, 2013). Within the nervous system, white matter fiber tracts, spinal nerves, and the cerebellum are affected (Lamarche, Lemieux, & Lieu, 1984). FRDA is the most common inherited ataxia presenting with early onset of clinical manifestations (Koeppen, 2011). So far, no effective treatment has been found that can stop or reverse the progress of FRDA. Therefore, it is important to assist the development of efficient medication by finding reliable imaging biomarkers that reflect the disease status and progress in FRDA patients.

An extensive body of literature of cerebral abnormalities in FRDA exists (Selvadurai, Harding, Corben, & Georgiou‐Karistianis, 2018) in which functional and structural cerebral and cerebellar white and gray matter changes are assessed: Volumetric, voxel‐based morphometry (VBM), and tract‐based spatial statistics (TBSS) magnetic resonance imaging (MRI) studies have shown a significant atrophy of the cerebral and cerebellar cortex in FRDA patients (Cocozza et al., 2020; Selvadurai et al., 2016) and the dentate nuclei (DN; Solbach et al., 2014; Stefanescu et al., 2015), a degeneration of white matter tracts (Della Nave et al., 2008; Pagani et al., 2010; Selvadurai et al., 2020), as well as a significant correlation of those findings with disease severity or certain symptoms (Akhlaghi et al., 2011) such as the impairment of the visuospatial functions (Cocozza et al., 2020). Another study including VBM and TBSS analyses found atrophy in several parts of the brainstem, and cerebellum, but did not observe any focal signal abnormalities on proton‐density and on *T*_2_‐weighted images (Pagani et al., 2010). A recent study (Selvadurai et al., 2020), that used multiple diffusion imaging‐based metrics as well as magnetization transfer imaging to assess microstructural and *T*_1_‐weighted imaging to assess macrostructural white matter changes found extensive disruption of cerebello‐cerebral and intracerebral white matter tracts. Quantitative methods such as quantitative susceptibility mapping (QSM; Ward et al., 2019) and *T*_2_ mapping (da Silva et al., 2014) seemed to provide useful information on longitudinal change of disease severity that correlated with longitudinal change in DN magnetic susceptibility. Most conclusive among several studies is the relation of disease severity and degenerative changes in the superior cerebellar peduncles and in peridentate white matter as well as the DN pathology which is an established observation in FRDA.

Ultra‐high field MRI has been shown to outperform conventional clinical MRI in the delineation of very small anatomical structures (Deistung, Schafer, Schweser, Biedermann, Gullmar, et al., 2013; Straub et al., 2019), and in the detection of small pathological changes such as in epilepsy (Zeineh et al., 2009) and micro‐hemorrhage (Theysohn et al., 2011) due to the higher resolution as well as the higher contrast‐to‐noise ratios available at higher field strengths (Ladd et al., 2018). To the best of our knowledge, no study has assessed whether quantitative MRI other than diffusion tensor imaging (DTI), magnetization transfer ratio, or spectroscopy (Blair et al., 2019) could provide complementary or additional information to evaluate the current stage and the course of Friedrich's ataxia. Compared to 3 T MRI, it has been shown that 7 T MRI can provide contrast‐to‐noise ratios up to almost fourfold higher for certain structures in the brainstem (Straub et al., 2019). Utilizing the higher contrast‐to‐noise ratio available at ultra‐high field, this study investigates the degeneration of fiber tracts throughout the brainstem as well as of the DN, the red nuclei (RN), and the substantia nigra (SN) in FRDA with quantitative MR parameters (susceptibility, diffusion anisotropy, and *R*_2_ and *R*_1_ relaxometry) by assessing a patient and a control group, and compares its results with a VBM analysis of the same cohorts. QSM was chosen as a contrast due to its ability to quantify tissue iron content (Langkammer, Schweser, et al., 2012) and its sensitivity to myelin (Lee et al., 2012). *R*_1_ and *R*_2_ relaxometry was used since its known to be influenced by myelin and iron (Harkins et al., 2016; Koenig, 1991; Langkammer, Krebs, et al., 2012; MacKay et al., 2006; Ogg & Steen, 1998). DTI has long been utilized to assess white matter fiber integrity (Chanraud, Zahr, Sullivan, & Pfefferbaum, 2010), and was therefore also included as a quantitative MRI parameter. Whether the used quantitative MRI methods provide sufficiently large effects to reveal differences between FRDA patients and healthy controls as well as correlations with disease characteristics at clinical field strengths are beyond the scope of this study and left to further investigations.

## 2 METHODS AND MATERIALS

### 2.1 Patient and control cohorts

This study was conducted in accordance with the Declaration of Helsinki. Institutional review board approval was obtained and all subjects provided written informed consent. Inclusion criteria were diagnosis of FRDA, physical and mental capability of providing informed consent and participating in the study. Exclusion criteria involved pregnancy and contraindications for 7 T MRI. As FRDA is a rare disease, the inclusion of 10 patients was the goal to achieve a sufficiently large patient cohort for the analyses. Ten genetically confirmed FRDA patients (mean age 37 ± 14 years; four female) and 10 age‐ and sex‐matched healthy controls (mean age 37 ± 14 years; four female) were included in the study. Patients were recruited at the Department for Neurology, Medical University of Innsbruck, Austria, and MR images were generated at the DKFZ Heidelberg, Germany. All participants finished the study. Nine patients had homozygous GAA triplet expansions and one patient was heterozygous (one GAA triplet expansion and one point mutation). For eight patients, length of GAA triplet repeats was available. Table 1 summarizes the patient cohort.

**TABLE 1:** Patient characteristics including patient age, sex, age at disease onset in years, and the number of GAA repeats

Patient number	Age in years	Age of disease onset in years	GAA1 repeats	GAA2 repeats	Sex
1	22	12	>700	>700	Male
2	49	28	250	250	Female
3	62	22	120	150	Male
4	46	34	350	350	Male
5	50	27	350	350	Female
6	34	20	500	600	Female
7	31	17	400	950	Male
8	18	15	700	970	Female
9	37	17	–	n.a.	Male
10	23	15	–	–	Male

Abbreviations: GAA1/GAA2, GAA repeats; n.a., not applicable (point mutation).

### 2.2 Data acquisition

Data were acquired at a whole‐body 7 T system (Magnetom 7 Tesla) with an 8Tx/32Rx‐channel head coil (Nova Medical Inc., Wakefield, MA, USA) by use of an in‐house‐constructed butler matrix. A monopolar three‐dimensional multi‐echo gradient echo (GRE), a two‐dimensional (2D) RESOLVE (readout segmentation of long variable echo‐trains; Heidemann et al., 2010; Porter & Heidemann, 2009) with 11 shots in stimulated acquisition mode (STEAM; Merboldt, Hanicke, & Frahm, 1991; Tanner, 1970) with mixing time 50 ms and with b‐values 50 and 800 s/mm^2^ and 20 diffusion directions, an EPI (echo planar imaging) factor of 68, echo spacing of 0.32 ms, and fat saturation, and a vendor‐provided MP2RAGE (magnetization prepared 2 rapid acquisition gradient echo; Marques et al., 2010) sequence were acquired using the same parameters as in a previous study (Straub et al., 2019). The RESOLVE sequence is equipped with navigator‐based reacquisition which was used for the acquisition of the DTI data. For dictionary‐based *T*_2_ mapping, a 2D multi‐echo turbo spin echo (ME‐TSE) with turbo factor 5 and a turbo flash sequence for *B*_1_ mapping (Chung, Kim, Breton, & Axel, 2010) were acquired as well. All sequences were acquired in strictly transversal orientation with phase‐encoding direction right–left. Sequence parameters are summarized in Table 2. Patients and controls received anonymized subject identifications numbers (IDs), and all MR images contained theses IDs only.

**TABLE 2:** Magnetic resonance imaging measurement parameters

	Resolution in mm^3^, matrix size	TR in ms	TE in ms	TI in ms	FA in °	BW in Hz/px	GRAPPA (factor/ref. lines)	Partial Fourier (slice, phase)	Acquisition time min:sec
ME‐GRE	0.47 × 0.47 × 0.47	38	10.5, 21, 31.5	–	12	140	2/36	6/8, 7/8	18:09
	464 × 348 × 192								
RESOLVE	1.2 × 1.2 × 2.4	2700	59, 99	–	180	631	2/68	–	23:55
	176 × 142 × 14								
ME‐TSE	0.49 × 0.49 × 2.0	7400	15, 88, 161,235	–	120	130	3/27	–	13:21
	448 × 336 × 30								
MP2RAGE	0.69 × 0.69 × 0.69	5000	4.01	900, 2750	4, 5	240	3/43	6/8, 7/8	8:08
	320 × 240 × 192								
Turbo flash (B_1_)	1.15 × 1.15 × 4.0	3000	2.36	–	7	490	–	–	1:32
	192 × 144 × 15								

Abbreviations: BW, bandwidth; GRAPPA, generalized autocalibrating partially parallel acquisitions; Hz, Hertz; ME‐GRE, multi‐echo gradient echo; ME‐TSE, multi‐echo turbo spin echo; min:sec, minutes:seconds; MR2RAGE, magnetization prepared 2 rapid acquisition gradient echo; ms, milliseconds; px, pixel; ref., reference; RESOLVE, readout segmentation of long variable echo‐trains;TE, echo time; TI, inversion time; TR, repetition time.

### 2.3 Data processing

#### 2.3.1 Quantitative susceptibility mapping

For susceptibility map generation, single‐channel data were combined using the computationally efficient combination of multi‐channel phase data from multi‐echo acquisitions (ASPIRE; Eckstein et al., 2018) on the scanner and echo‐wise unwrapped with Laplacian‐based phase unwrapping (Li, Avram, Wu, Xiao, & Liu, 2014; Li, Wu, & Liu, 2011; Wu, Li, Guidon, & Liu, 2012). After background field removal with the variable‐kernel sophisticated harmonic artifact reduction for phase data (V‐SHARP; Li et al., 2014; Wu, Li, Guidon, et al., 2012) method (kernel size up to 12 mm), all echoes were averaged (Wu, Li, Avram, Gho, & Liu, 2012), and a dipole inversion was performed using the streaking artifact reduction for QSM (STAR‐QSM) algorithm (Wei et al., 2015). Background field removal required a brain mask which was generated with FSL‐BET (Brain Extraction Tool; Smith, 2002), and manually corrected in the Medical Imaging Interaction Toolkit (MITK; Maleike, Nolden, Meinzer, & Wolf, 2009; Nolden et al., 2013). Volumes of interest (VOIs) for cerebrospinal fluid (CSF) in the atria of the lateral ventricles were manually drawn in MITK and used to reference susceptibility maps (Straub et al., 2017). Except for the coil combination, all processing steps were performed in Matlab (Matlab, R2017b, MathWorks, Natick, USA).

#### 2.3.2 Relaxometry and diffusion tensor imaging

The longitudinal relaxation rate *R*_1_ was calculated as *R*_1_ = 1/*T*_1_ from the vendor‐provided *T*_1_ maps that were acquired with the MP2RAGE sequence. The vendor‐provided diffusion fractional anisotropy maps were used for the diffusion data evaluation. Maps of the transverse relaxation rate *R*_2_ = 1/*T*_2_ were calculated from ME‐TSE data and *B*_1_ maps using a dictionary‐based method (Emmerich et al., 2019): A dictionary for the echo intensities was computed for the ME‐TSE sequence according to the sequence parameters (Table 2) using a combination of Bloch simulations to compute the slice profiles and subsequently extended phase graph model (Weigel, 2015) simulations to compute the resulting signal intensities. The measured signal intensities and *B*_1_ maps were then fitted to this precalculated dictionary to obtain *T*_2_ values.

### 2.4 Data analysis

#### 2.4.1 Volumes of interest generation

**FIGURE 1:**
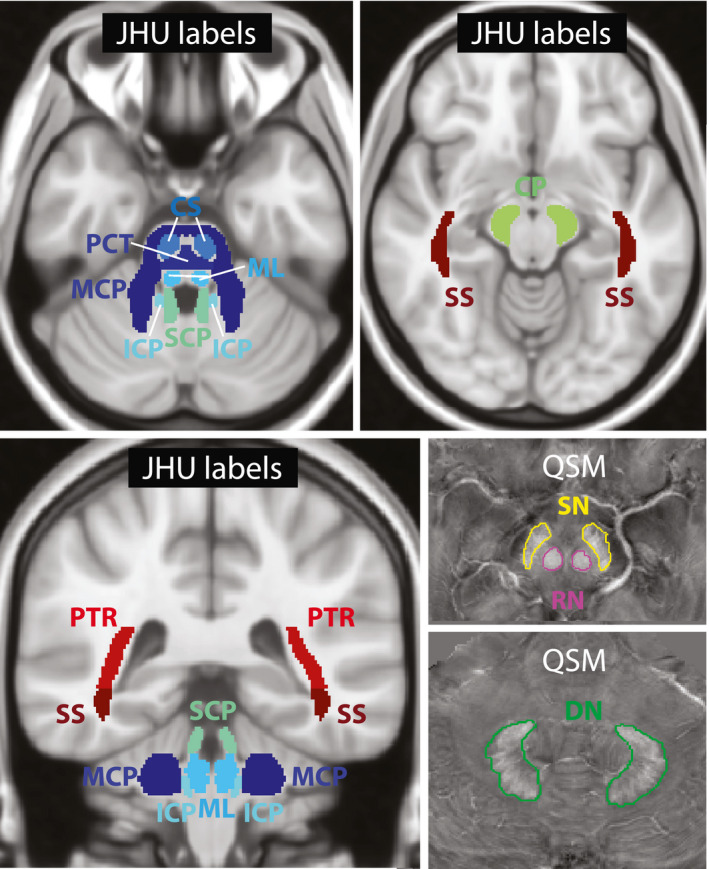
Overview of the volumes of interest. JHU atlas labels in MNI space that were used as volumes of interest for the middle cerebellar peduncle (MCP), pontine crossing tract (PCT), corticospinal tract (CS), medial lemniscus (ML), inferior cerebellar peduncle (ICP), superior cerebellar peduncle (SCP), cerebral peduncle (CP), posterior thalamic radiation (PTR; including optic radiation), sagittal stratum (SS; including inferior longitudinal fasciculus and inferior fronto‐occipital fasciculus), and manually drawn volumes of interest for substantia nigra (SN), red nuclei (RN), and dentate nuclei (DN) on susceptibility maps (QSM) are shown [Color figure can be viewed at wileyonlinelibrary.com]

Volumes of interest (Figure 1) were generated for nine fiber tracts (middle cerebellar peduncle—MCP, pontine crossing tract—PCT, corticospinal tract—CS, medial lemniscus—ML, inferior cerebellar peduncle—ICP, superior cerebellar peduncle—SCP, cerebral peduncle—CP, posterior thalamic radiation (including the optic radiation)—PTR, sagittal stratum (including the inferior longitudinal, fasciculus, and the inferior fronto‐occipital fasciculus)—SS) by registering JHU‐ICBM‐DTI‐81 white matter labels (Mori et al., 2008) to the MP2RAGE data of this study with FSL‐FLIRT (Jenkinson & Smith, 2001) and FSL‐FNIRT (Andersson, Jenkinson, & Smith, 2010) using the FSL MNI152_T1_0.5mm template. For DN, RN, and SN, bilateral VOIs were generated manually on the susceptibility maps using MITK including all slices on which the structures were morphologically visible for each patient/control individually. All VOIs were subsequently registered to the other imaging contrasts with FSL‐FLIRT and automatically corrected for registration errors by thresholding to exclude CSF pixels in the case of *R*_1_, *R*_2_, and FAmaps to eliminate voxels from the VOIs for which $R_{1}<\frac{1}{3000}\frac{1}{\mathrm{ms}}$, $R_{2}<\frac{1}{170}\frac{1}{\mathrm{ms}},$ and FA < 300, respectively. In the case of susceptibility maps, a CSF mask that was generated with FSL‐FAST (Zhang, Brady, & Smith, 2001) was used for automatic correction. A manual VOI assessment ensued, as well as a correction in cases in which pixels were included in the VOI that clearly did not belong to the corresponding structure, i.e. pixels that were wrongly positioned in the CSF or belonged to a different anatomical structure. As the DTI data only covered an approximately four centimeter slab, not all fiber tracts were feasible for DTI analysis, the brain nuclei were not included in DTI analysis as well.

#### 2.4.2 Voxel‐based morphometry

VBM (Ashburner & Friston, 2000; Mechelli, Price, Friston, & Ashburner, 2005) was performed using the MP2RAGE data and Statistical Parametric Mapping (SPM12, https://www.fil.ion.ucl.ac.uk/spm/software/spm12/) in Matlab. Following (Ashburner, 2010), first, grey matter, white matter, CSF, skull, and head were segmented using default parameters and generating the input data for the next step. Then, a template was generated from all controls and patients using the “Diffeomorphic Anatomical Registration Using Exponentiated Lie Algebra” (DARTEL) algorithm (Ashburner, 2007) with default parameters. Finally, DARTEL tools were used to normalize white matter images to the Montreal Neurological Institute (MNI) space which included smoothing with an 8‐mm Gaussian kernel, spatially normalizing, and scaling by the Jacobian determinant.

#### 2.4.3 Statistical analysis

All statistical analyses were performed in Matlab, and with SPM12 for the general linear model analysis for VBM evaluation. Mean susceptibility values, relaxation rates, and mean diffusion fractional anisotropy (FA) were calculated. Differences between the patients and the healthy controls were assessed using the analysis of covariance (ANCOVA) with age and sex as covariates, and effect size of the group differences between healthy controls and patients was calculated using Cohen's *d*. Correlations with FRDA characteristics as well as with patient age (Table 1) were assessed using the Spearman correlation coefficient. *p*‐Values of less than 0.01 were considered statistically significant. To analyze statistically significant differences in white matter volume from the VBM evaluation between the patient and the control group, a two‐sided t‐test was performed in SPM12 with age and sex as covariates, first uncorrected with a statistical threshold of 0.001, and secondly using the SPM12 family‐wise error correction to correct for multiple comparisons with a statistical threshold of 0.05. No data were removed prior to analysis. Data processing was performed blinded to disease characteristics.

## 3 RESULTS

### 3.1 Differences between FRDA patients and controls

In Table 3, the effect size for the differences between the patient and the control group is given by Cohen's *d*. Largest effect size (above 2) is observed for *R*_2_ relaxometry of the RN and for diffusion fractional anisotropy of the SCPs. For mean susceptibility values of the DN, and RN, for mean *R*_1_ values of the ICPs and SCPs, and RN, for mean *R*_2_ values of the SCPs, CPs, RN, and SN, as well as for mean diffusion fractional anisotropy of the CS, the ICPs, CPs, the effect size is larger than 1. For mean *R*_1_ values of five other fiber tracts (MCP, ML, CP, PTR, and SS), mean *R*_2_ values of three other fiber tracts (MCP, ML, and PTR), and diffusion fractional anisotropy of three other fiber tracts (MCP, PCT, and ML), the effect size is larger than 0.5.

**TABLE 3:** Effects size (Cohen's *d*) of group differences

	QSM	*R*_1_	*R*_2_	FA
MCP		0.65	0.66	0.53
PCT				0.55
CS				1.18
ML		0.76	0.95	0.57
ICP		1.06		1.77
SCP		1.46	1.22	2.28
CP		0.61	1.70	1.06
PTR		0.71	0.83	n.a.
SS		0.60		n.a.
DN	1.46			n.a.
RN	1.62	1.61	2.91	n.a.
SN			1.16	n.a.

	0.5–0.99	1–1.99	2–2.99	

Abbreviations: CP, cerebral peduncle; CS, corticospinal tract; DN, dentate nuclei; FA, fractional anisotropy; ICP, inferior cerebellar peduncle; MCP, the middle cerebellar peduncle; ML, medial lemniscus; PCT, pontine crossing tract; PTR, posterior thalamic radiation (including optic radiation); QSM, quantitative susceptibility mapping; RN, red nuclei; SCP, superior cerebellar peduncle; SN, substantia nigra; SS, sagittal stratum (including inferior longitudinal fasciculus and inferior fronto‐occipital fasciculus).

**FIGURE 2:**
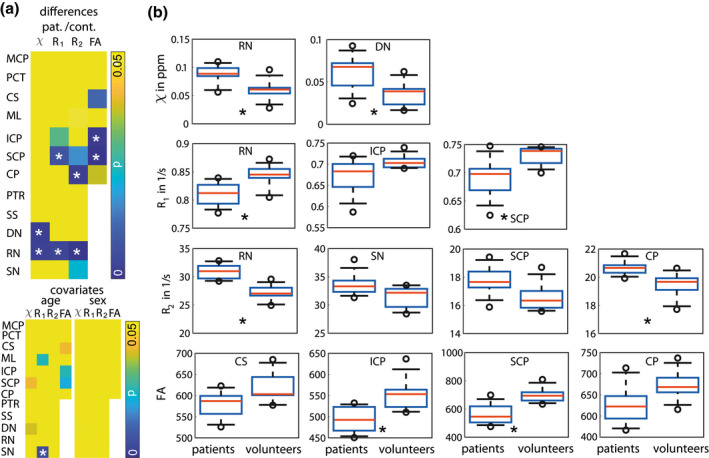
Significant differences between healthy controls and patients indicated with a star. (a) *p*‐Values of ANCOVA for differences and covariates (age and sex) in susceptibility values (χ), *R*_1_ and *R*_2_ values, and diffusion fractional anisotropy (FA) of mean VOI values of the middle cerebellar peduncle (MCP), pontine crossing tract (PCT), corticospinal tract (CS), medial lemniscus (ML), inferior cerebellar peduncle (ICP), superior cerebellar peduncle (SCP), cerebral peduncle (CP), posterior thalamic radiation (PTR; including optic radiation), sagittal stratum (SS; including inferior longitudinal fasciculus and inferior fronto‐occipital fasciculus), dentate nuclei (DN), red nuclei (RN), and substantia nigra (SN). For PTR, SS, DN, RN, and SN, FA values were not available from all patients and controls due to insufficient brain coverage of the field of view. (b) Box plots of mean susceptibility values, mean *R*_1_, mean *R*_2_, and mean diffusion fractional anisotropy (b) for all patients and healthy controls. Only box plots for low *p*‐values (see a) are shown. The whiskers represent the 9th and the 91st percentile [Color figure can be viewed at wileyonlinelibrary.com]

In five brain regions, which coincide with the regions of largest effect size (Table 3, Cohen's *d* 1.46 or larger), statistically significant differences (indicated by white stars) were found between healthy controls and patients. Figure 2a summarizes these differences as well as the covariates (age and sex) by showing the *p*‐values of the ANCOVA. In Figure 2b, box plots for the VOIs and contrasts, for which *p*‐values were low (≪0.05), are shown and significant differences are indicated with a black star. The corresponding region is indicated within each box plot. Susceptibility mapping revealed significant differences between healthy controls and patients for the DN and RN for which susceptibility values where higher in patients. In *R*_1_ maps, values for RN were significantly lower in patients and significantly higher in SCP. *R*_2_ mapping showed significant differences between controls and patients for RN, and for one fiber tract (CP), where *R*_2_ was significantly lower/higher. Diffusion fractional was significantly lower in patients in two fiber tracts (ICP and SCP). For SN, there was a significant correlation with age.

**FIGURE 3:**
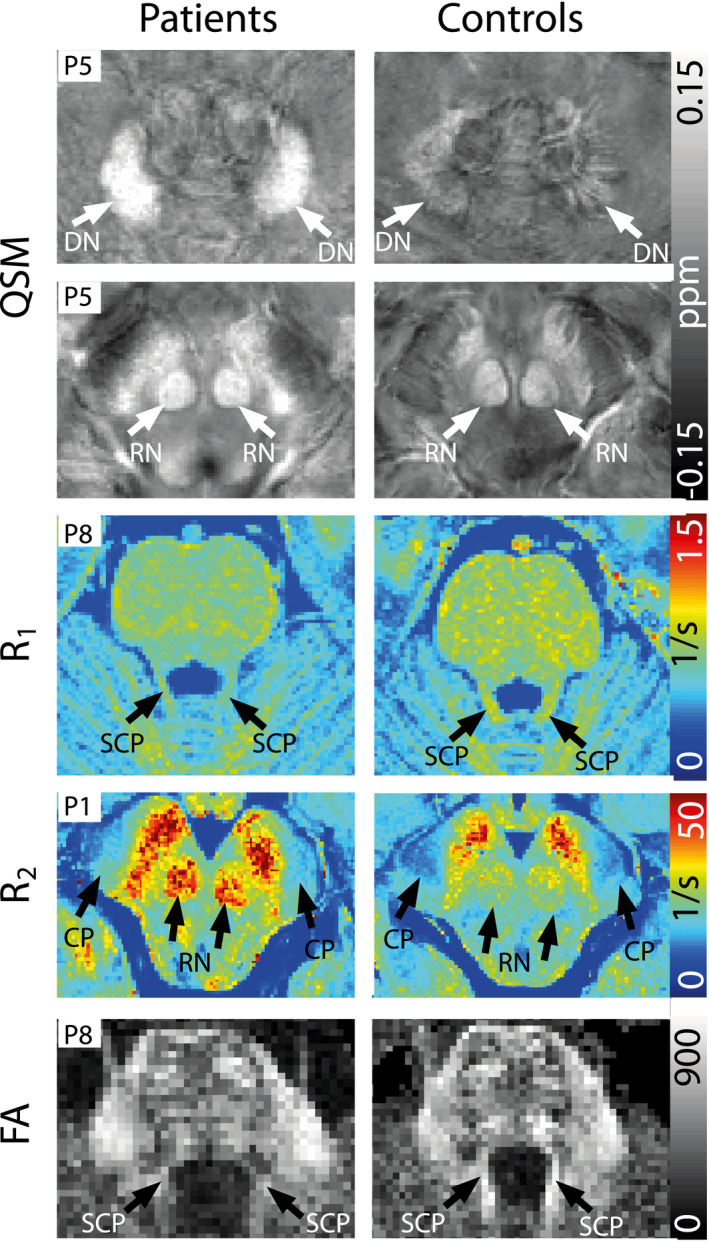
Representative axial slices of susceptibility, *R*_1_, *R*_2_, and fractional anisotropy maps (top to bottom) from four different patients and controls. The patient number is indicated in each slice. On susceptibility maps, arrows point to dentate nuclei (DN), and red nuclei (RN), on the *R*_1_ map to superior cerebellar peduncles (SCP), on the *R*_2_ map to the red nuclei (RN), and the cerebral peduncle (CP), and on the fractional anisotropy map arrows point to superior cerebellar peduncles (SCP) [Color figure can be viewed at wileyonlinelibrary.com]

Moreover, the differences revealed by the statistical analysis are also visually recognizable. In Figure 3, representative slices of susceptibility, *R*_1_ and *R*_2_ maps, as well as fractional anisotropy maps from diffusion‐weighted imaging are shown for brain regions for which significant differences between healthy controls and patients were found. Images from different patients (indicated by patient number P) and age‐/sex‐matched controls are shown. On susceptibility maps, conspicuous differences in DN (first row) and RN (second row) can be observed with a higher susceptibility on the patient images compared to healthy controls. On *R*_1_ maps, higher *R*_1_ relaxation rates are visible in the SCP (third row). Higher *R*_2_ relaxation rates can be seen in the RN, and the CP (fourth row) on *R*_2_ maps for the patient data. On fractional anisotropy maps, a lower fractional anisotropy can be observed for the SCP (fifth row) in the patient data.

### 3.2 Voxel‐based morphometry

**FIGURE 4:**
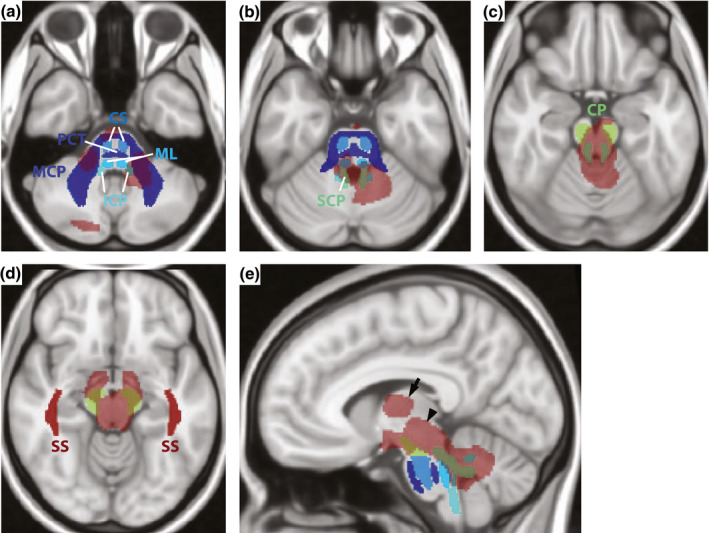
Agreement of voxel‐based morphometry and volume of interest‐based analysis. Suprathreshold *t*‐values (*p* < 0.001, uncorrected) show regions of significant white matter atrophy overlaid on three representative axial (a–d) and one sagittal slice (e) of the 0.5 mm MNI_T1 space template. JHU labels for the middle cerebellar peduncle (MCP), the pontine crossing tract (PCT), the corticospinal tract (CS), the medial lemniscus (ML), the inferior cerebellar peduncle (ICP), the superior cerebellar peduncle (SCP), the cerebral peduncle (CP), the posterior thalamic radiation (PTR), and the sagittal stratum (SS) are overlaid as well. The arrow indicates the region of the thalamus and the arrow head indicates the midbrain [Color figure can be viewed at wileyonlinelibrary.com]

Compared to the age‐ and sex‐matched controls, statistically significant, white matter atrophy was found in the patient cohort in the uncorrected t‐test analysis (*p* < 0.001). Figure 4 shows suprathreshold t‐values (transparent red) representing the significant white matter atrophy in the patient cohort, and the JHU‐ICBM‐DTI‐81 white matter labels, which were used in this study, overlaid on the 0.5 mm MNI space template. Except for the VOIs of SS and PTR, suprathreshold t‐values partially overlap with all white matter VOIs used in this study. A large overlap can be observed for the cerebellar peduncles (ICP and SCP). Moreover, suprathreshold t‐values were found in the regions of the RN and SN (VOIs not shown), in peridentate white matter, as well as in parts of the thalamus (Figure 4e, arrow), and in large parts of the midbrain (Figure 4e, arrow head). For the corrected t‐test analysis, no statistically significant clusters were found.

### 3.3 Correlation analysis

**FIGURE 5:**
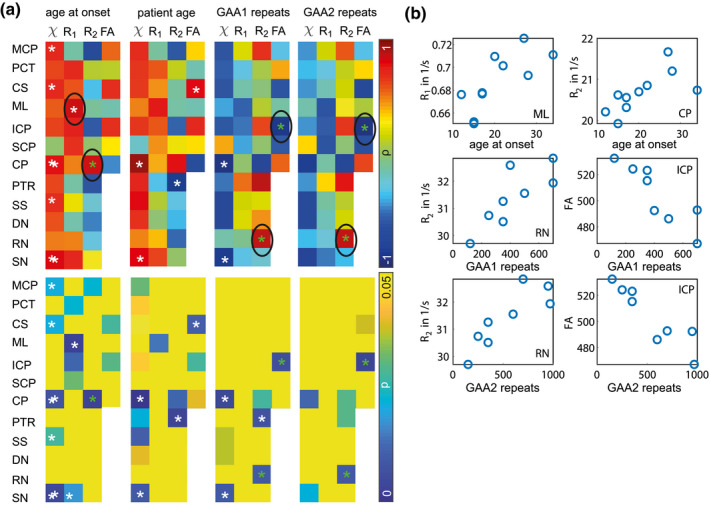
Correlations between quantitative MRI parameters and FRDA patients' disease characteristics as well as with patient age. (a) Correlation coefficients (ρ, first row) and *p*‐values (*p*, second row) for correlations between mean volumes of interest (VOIs) values for the different imaging contrasts (susceptibility χ, relaxation rates *R*_1_ and *R*_2_, diffusion fractional anisotropy FA) and age at onset (first column), patient age (second column), GAA1 repeats (third column), and GAA2 repeats (fourth column). White stars indicate significant correlations, green stars indicate that for this contrast/VOI combination, additionally significant differences between patients and healthy controls have been found (see Figure 2). Correlations for contrast/VOI combinations, for which no correlation with patient age was found, are marked with a black circle. (b) Scatterplots for the contrasts and VOIs for which significant correlations with disease characteristic, but not with patient age were found [Color figure can be viewed at wileyonlinelibrary.com]

In Figure 5, the correlations between the different imaging contrasts and disease characteristics are visualized. Figure 5a shows the correlation coefficients and *p*‐values for the correlations between age at disease onset, patient age, GAA1 repeats, GAA2 repeats (Table 1) and mean susceptibility, *R*_1_, *R*_2_, fractional anisotropy values in the different VOIs. Statistically significant correlations are indicated by white stars; green stars indicate that additionally significant differences have been found for the respective contrast/VOI combination. For the significant correlations between mean susceptibility values and disease characteristics, a significant correlation with patient age was revealed as well, possibly indicating a bias. The significant correlations between disease characteristics and contrast/VOI combinations for which no significant correlation with patient age was found are indicated with black circles. Figure 5b shows the scatterplots of mean *R*_1_ values in ML, mean *R*_2_ values in CP and RN, and mean FA values in ICP. All correlation coefficients and *p*‐values are given in Table S1.

## 4 DISCUSSION

In this study, statistically significant (*p* < 0.01, no correction for multiple comparisons) differences between a group of 10 FRDA patients and a group of age‐ and sex‐matched healthy controls were found in five (CP, DN, ICP, SCP, and RN) out of twelve investigated structures of the brainstem, and the cerebellum using quantitative MRI (susceptibility, relaxation rates (*R*_2_,* R*_1_), diffusion fractional anisotropy) at 7 Tesla. It was also shown that the effect size of group differences for susceptibility, *R*_2_, *R*_1_, and fractional anisotropy values between healthy controls and patients were medium to large (0.53–2.91) for several fiber tracts and brain nuclei, indicating that the used methods in the investigated structures could be useful biomarkers in larger studies. In the present study, only VOI/contrast combinations with an effect size of 1.46 (Cohen's *d*) or larger for group differences provided significant differences between patients and controls. Moreover, using VBM, statistically significant white matter atrophy was found within regions of the brainstem, midbrain, and the cerebellum that partially overlapped with brain regions for which significant differences between healthy controls and patients were found in the VOI‐based quantitative MRI evaluation part of this study. The largest overlap was observed for SCP for which significant differences between patients and healthy controls were found using *R*_1_ relaxometry and diffusion‐weighted imaging indicating white matter damage in patients. The atrophy of SCP shown in FRDA previously (Akhlaghi et al., 2011; Della Nave et al., 2008; Pagani et al., 2010) is in accordance with the findings of this study. Moreover, SCP atrophy as well as the atrophy of the peridentate white matter were suggested to correlate with disease symptoms (Akhlaghi et al., 2011; Pagani et al., 2010). Using VBM, atrophy in FRDA patients was also found in the central portion of the medulla oblongata, the dorsal upper pons, the central portion of the midbrain, the medial portion of the right CP, and optic chiasm (Pagani et al., 2010) which partly coincides with the VBM findings of this study. Atrophy of the DN (Solbach et al., 2014) and the RN (Rezende et al., 2019) as well as higher iron content in the DN have been reported in FRDA (Koeppen et al., 2007; Waldvogel, van Gelderen, & Hallett, 1999). These findings are in accordance with the findings of this study, in which higher susceptibility values, higher *R*_2_ values, and lower *R*_1_ values were found in the RN as well as higher susceptibility values in the DN. Iron accumulation or iron redistribution and DN atrophy are well‐known features in the cerebral pathology of FRDA (Selvadurai et al., 2018), and pathological changes in RN are supposed to be due to their tight link to the DN, and consequently to the loss or reduction of input through the SCP (Akhlaghi et al., 2014; Habas, Guillevin, & Abanou, 2010; Nioche, Cabanis, & Habas, 2009) which is in accordance with the findings of this study in which pathologic differences were found in DN (with QSM), RN (with QSM, *R*_1_ and *R*_2_ relaxometry), and SCP (with *R*_1_ relaxometry and fractional anisotropy). Even though a dictionary‐based *R*_2_ mapping method was used that compensates for *B*_1_ field inhomogeneities, very low *B*_1_ in parts of the cerebellum might affect *R*_2_ in the DN. This could explain why no significant differences between patients and controls were found in DN in contrast to the findings of this (da Silva et al., 2014) 3 T study. Gradient echo techniques such as susceptibility mapping are much less affected by *B*_1_ field inhomogeneities that occur particularly strong at ultra‐high field. However, susceptibility values calculated with standard QSM methods depend on fiber orientation (Aggarwal, Kageyama, Li, & van Zijl, 2016), which might be the reason why no significant differences were found with QSM in this study. Moreover, this study evaluated if significant correlations of quantitative MRI parameters of the investigated VOIs and disease characteristics were present. For all MRI contrasts significant correlations with one or more disease characteristics were found. Mean *R*_1_ values within the ML positively correlated with age at disease onset, as well as mean *R*_2_ values within the CP which could indicate more white matter damage in the patients with the earlier disease onset agreeing with current literature (Selvadurai et al., 2020). Mean *R*_2_ values in the RN positively correlated with GAA1 and GAA2 repeats which are in accordance with the relation of GAA repeat length and frataxin levels (Sacca et al., 2011). Fractional anisotropy in the ICP negatively correlated with GAA1 and GAA2 repeats which also agrees with findings in the literature (Selvadurai et al., 2020) that found a correlation of ICP volume and GAA1 repeats. For the significant susceptibility/VOI correlation pairs, a significant correlation with patient age was revealed as well. Magnetic susceptibility measured with QSM is known to be sensitive for age‐dependent iron accumulation within the brain nuclei (Acosta‐Cabronero, Betts, Cardenas‐Blanco, Yang, & Nestor, 2016; Hallgren & Sourander, 1958; Zhang et al., 2018). In this study, this could mask any disease‐related changes within the patient group as higher susceptibility values due to iron accumulation could be related to higher patient age or to increased disease severity (that is, longer GAA repeats/earlier age at onset) which cannot be differentiated with the studied cohort. As the control cohort was age‐matched, significant differences could very well be observed and results consequently did not suffer from an age‐bias.

This study has several limitations. The low number of included patients limits achievable statistical significance. Since FRDA is a rare disease, studies are often rather small (Akhlaghi et al., 2011; Pagani et al., 2010), however, there exist studies using 3 T MRI that include approximately 30 FRDA patients (Selvadurai et al., 2020). Moreover, the patient cohort was relatively inhomogeneous consisting of three young patients (between 18 and 23 years) with early onset (before the age of 25 years) of FRDA, three patients in their 30s as well as one patient aged 62 with early onset, and three patients older than 45 years with rather late onset (older than 25 years). Sex was also only considered as covariate, and sex‐related differences were not assessed separately due to the rather low patient number; however, all patients and controls were age‐ and sex‐matched. To further evaluate the value of quantitative MRI for assessing disease status as well as progression and correlation with clinical parameters, studies including larger homogeneous cohorts at high or ultra‐high field might be helpful. Moreover, due to a stronger presence of field inhomogeneities at ultra‐high field, DTI is even more prone to artifacts compared to clinical field strength, therefore, a readout‐segmented EPI sequence with navigator‐based reacquisition was chosen at the expense of longer acquisition times. To avoid prohibitively long acquisition times, only 20 diffusion directions were used as well as a lower coverage of the brain. JHU‐ICBM‐DTI‐81 white matter labels were co‐registered to the MP2RAGE, as these possessed the largest brain coverage, data of this study and then subsequently co‐registered to all other contrasts which could be prone to registration errors despite the automatic and manual corrections applied to the VOIs. The fact that the JHU‐ICBM‐DTI‐81 is in standard space, however, not a standard space template might further be a source of error. For the VBM analysis, no normalization with total intracranial volume was used since the MP2RAGE sequence did not cover the entire brain in all patients. Finally, VBM itself has received critique concerning its reliability and precision (Ashburner & Friston, 2001; Bookstein, 2001; Michael, Evans, & Moore, 2016; Scarpazza, Tognin, Frisciata, Sartori, & Mechelli, 2015).

Although the present results are promising, access to ultra‐high‐field MRI is still limited as 7 T MRI scanners are currently more expensive and less available in clinical and research centers, whereas 3 T can routinely be applied. However, 7 T MRI scanners have various advantages in comparison to the routine MRI: Higher contrast‐to‐noise and signal to‐noise ratios at ultra‐high field allow for higher possible spatial resolutions, and consequently result in higher sensitivity to tissue changes and anatomical details, which thus produces clearer tissue boundaries and helps visualize small structures *in vivo* within reasonable scan time (Deistung, Schafer, Schweser, Biedermann, Turner, et al., 2013; Karamat, Darvish‐Molla, & Santos‐Diaz, 2016; Straub et al., 2019; Thomas et al., 2008). This study aimed at investigating quantitative MRI parameters which could assist to gain further insight in the mechanisms of the pathology in FRDA. To further evaluate the added value of the 7 T study over 3 T MRI for clinical routine application, a consecutive study is planned in the same study cohort. Replication of the present study results would facilitate their application at clinical field strengths, which is, however, beyond the scope of this study and therefore left to further investigations.

## 5 CONCLUSION

It was shown in this study that two independent analyses, a VOI‐based evaluation of quantitative MRI data and a VBM analysis, provided overlapping results. Susceptibility mapping provided significant differences between FRDA patients and controls, as well as* R*_1_, *R*_2_ relaxometry and diffusion imaging for two brain nuclei and three fiber tracts. Moreover, positive results on correlations with disease characteristics were found, indicating that these quantitative MRI parameters could provide more detailed information than volumetric assessment alone, and may assist the search for effective treatments.

## DECLARATION OF TRANSPARENCY

The authors, reviewers and editors affirm that in accordance to the policies set by the *Journal of Neuroscience Research*, this manuscript presents an accurate and transparent account of the study being reported and that all critical details describing the methods and results are present.

## CONFLICT OF INTERESTS

The authors report no conflict of interest.

## AUTHOR CONTRIBUTIONS

*Conceptualization,* S.S., S.M., M.E.L., S.B., and E.G.; *Methodology,* S.S., S.M., M.E.L., S.B., and E.G.; *Software,* S.S., J.E., and K.S.D.; *Validation,* S.S., S.M., J.E., K.S.D., M.E.L., S.B., and E.G.; *Formal Analysis,* S.S., S.M., J.E., and K.S.D.; *Investigation,* S.S., S.M., E.I., W.N., and E.G.; *Resources,* S.S., S.M., M.E.L., S.B., and E.G.; *Data Curation,* S.S., S.M., E.I., W.N., and E.G.; *Writing – Original Draft,* S.S.; *Writing – Review & Editing,* S.M., J.E., E.I., W.N., K.S.D., M.E.L., S.B., and E.G.; *Visualization,* S.S., S.M., J.E., K.S.D., M.E.L., and S.B.; *Supervision,* M.E.L. and S.B.; *Project Administration,* S.S., S.M., E.I., W.N., and E.G.

### PEER REVIEW

The peer review history for this article is available at https://publons.com/publon/10.1002/jnr.24701.

## Supporting information

Table S1 All Spearman correlation coefficients ρ and *p*‐values for Figure 5 are provided when applicable for all investigated brain structures, middle cerebellar peduncle (MCP), pontine crossing tract (PCT), corticospinal tract (CS), medial lemniscus (ML), inferior cerebellar peduncle (ICP), superior cerebellar peduncle (SCP), cerebral peduncle (CP), posterior thalamic radiation (PTR; including optic radiation), sagittal stratum (SS; including inferior longitudinal fasciculus and inferior fronto‐occipital fasciculus), and manually drawn volumes of interest for substantia nigra (SN), red nuclei (RN), and dentate nuclei (DN) and all contrasts, susceptibility mapping (QSM), *R*_1_ and *R*_2_ relaxometry, and fractional anisotropy (FA). Statistically significant findings are marked green

Transparent Peer Review Report

Transparent Science Questionnaire for Authors

## Data Availability

The authors take full responsibility for the data, the analyses and interpretation, and the conduct of the research and have full access to all of the data, of which we have the right to publish any and all data in the absence of a sponsor. Anonymized data, not published in the article, will be shared on reasonable request from a qualified investigator.
