# Supplementary material for: Toward quantitative neuroimaging biomarkers for Friedreich's ataxia at 7 Tesla: Susceptibility mapping, diffusion imaging, R 2 and R 1 relaxometry
Source: J Neurosci Res. 2020 Jul 30;98(11):2219–31. doi: 10.1002/jnr.24701 (PMC7590084; doi:10.1002/jnr.24701)
Supplement: Supplementary file 1 — Table S1 All Spearman correlation coefficients ρ and p‐values for Figure 5 are provided when applicable for all investigated brain structures, middle cerebellar peduncle (MCP), pontine crossing tract (PCT), corticospinal tract (CS), medial lemniscus (ML), inferior cerebellar peduncle (ICP), superior cerebellar peduncle (SCP), cerebral peduncle (CP), posterior thalamic radiation (PTR; including optic radiation), sagittal stratum (SS; including inferior longitudinal fasciculus and inferior fronto‐occipital fasciculus), and manually drawn volumes of interest for substantia nigra (SN), red nuclei (RN), and dentate nuclei (DN) and all contrasts, susceptibility mapping (QSM), R 1 and R 2 relaxometry, and fractional anisotropy (FA). Statistically significant findings are marked green [file JNR-98-2219-s001.docx]

Table S1: Correlation coefficients and p-values for the correlations between quantitative MRI parameters and FRDA patients’ disease characteristics as well as with patient age.

| Age at onset | |  |  |  |  |  |  |  |  |
| --- | --- | --- | --- | --- | --- | --- | --- | --- | --- |
| $\rho$ | $\chi$ | R_1_ | R_2_ | FA | p | $\chi$ | R_1_ | R_2_ | FA |
| MCP | 0.71 | -0.13 | -0.71 | 0.54 |  | 0.021 | 0.712 | 0.022 | 0.105 |
| PCT | 0.62 | 0.71 | 0.10 | 0.12 |  | 0.058 | 0.022 | 0.789 | 0.750 |
| CS | 0.71 | 0.58 | -0.34 | 0.69 |  | 0.021 | 0.079 | 0.334 | 0.028 |
| ML | 0.57 | 0.85 | -0.10 | -0.15 |  | 0.087 | 0.002 | 0.776 | 0.674 |
| ICP | 0.63 | 0.76 | -0.59 | 0.68 |  | 0.052 | 0.011 | 0.075 | 0.030 |
| SCP | -0.01 | 0.68 | -0.01 | 0.29 |  | 0.973 | 0.032 | 0.973 | 0.412 |
| CP | 0.79 | 0.49 | 0.84 | -0.46 |  | 0.007 | 0.153 | 0.002 | 0.184 |
| PTR | 0.62 | -0.13 | -0.60 |  |  | 0.058 | 0.724 | 0.065 |  |
| SS | 0.69 | 0.23 | -0.23 |  |  | 0.028 | 0.519 | 0.519 |  |
| DN | 0.59 | -0.15 | -0.47 |  |  | 0.072 | 0.687 | 0.171 |  |
| RN | 0.49 | 0.49 | -0.27 |  |  | 0.147 | 0.153 | 0.443 |  |
| SN | 0.82 | 0.73 | 0.26 |  |  | 0.004 | 0.018 | 0.475 |  |
|  |  |  |  |  |  |  |  |  |  |
| Patient age | |  |  |  |  |  |  |  |  |
| MCP | 0.70 | -0.12 | -0.59 | 0.33 |  | 0.031 | 0.759 | 0.080 | 0.349 |
| PCT | 0.66 | 0.58 | 0.13 | -0.16 |  | 0.044 | 0.088 | 0.733 | 0.657 |
| CS | 0.65 | 0.45 | -0.12 | 0.79 |  | 0.049 | 0.191 | 0.759 | 0.010 |
| ML | 0.49 | 0.77 | -0.13 | -0.16 |  | 0.154 | 0.014 | 0.733 | 0.657 |
| ICP | 0.66 | 0.58 | -0.54 | 0.71 |  | 0.044 | 0.088 | 0.113 | 0.028 |
| SCP | -0.21 | 0.45 | 0.24 | 0.31 |  | 0.560 | 0.191 | 0.514 | 0.387 |
| CP | 0.95 | 0.41 | 0.78 | -0.67 |  | 0.000 | 0.247 | 0.012 | 0.039 |
| PTR | 0.73 | -0.33 | -0.87 |  |  | 0.021 | 0.349 | 0.003 |  |
| SS | 0.79 | 0.09 | -0.30 |  |  | 0.010 | 0.811 | 0.407 |  |
| DN | 0.67 | -0.03 | -0.38 |  |  | 0.039 | 0.946 | 0.279 |  |
| RN | 0.48 | 0.37 | -0.53 |  |  | 0.166 | 0.296 | 0.123 |  |
| SN | 0.81 | 0.62 | -0.01 |  |  | 0.008 | 0.060 | 1.000 |  |
|  |  |  |  |  |  |  |  |  |  |
| GAA1 repeats | |  |  |  |  |  |  |  |  |
| MCP | -0.60 | 0.19 | 0.67 | -0.31 |  | 0.122 | 0.652 | 0.077 | 0.445 |
| PCT | -0.57 | -0.30 | 0.06 | 0.41 |  | 0.151 | 0.461 | 0.894 | 0.313 |
| CS | -0.60 | -0.14 | -0.04 | -0.61 |  | 0.122 | 0.734 | 0.939 | 0.112 |
| ML | -0.41 | -0.55 | 0.10 | -0.13 |  | 0.313 | 0.163 | 0.829 | 0.756 |
| ICP | -0.59 | -0.49 | 0.61 | -0.89 |  | 0.132 | 0.216 | 0.112 | 0.005 |
| SCP | 0.41 | -0.41 | -0.01 | -0.58 |  | 0.313 | 0.313 | 0.985 | 0.142 |
| CP | -0.92 | -0.01 | -0.78 | 0.55 |  | 0.003 | 0.985 | 0.027 | 0.163 |
| PTR | -0.49 | 0.53 | 0.88 |  |  | 0.216 | 0.184 | 0.007 |  |
| SS | -0.76 | 0.17 | 0.19 |  |  | 0.037 | 0.693 | 0.652 |  |
| DN | -0.76 | 0.19 | 0.46 |  |  | 0.037 | 0.652 | 0.258 |  |
| RN | -0.14 | -0.08 | 0.87 |  |  | 0.734 | 0.851 | 0.009 |  |
| SN | -0.87 | -0.31 | -0.23 |  |  | 0.009 | 0.445 | 0.582 |  |
|  |  |  |  |  |  |  |  |  |  |
| GAA2 repeats | |  |  |  |  |  |  |  |  |
| MCP | -0.44 | 0.17 | 0.57 | -0.32 |  | 0.275 | 0.692 | 0.143 | 0.434 |
| PCT | -0.37 | -0.29 | 0.13 | 0.32 |  | 0.365 | 0.487 | 0.761 | 0.434 |
| CS | -0.44 | -0.17 | 0.17 | -0.75 |  | 0.275 | 0.692 | 0.692 | 0.038 |
| ML | -0.16 | -0.61 | 0.25 | 0.07 |  | 0.716 | 0.116 | 0.549 | 0.872 |
| ICP | -0.44 | -0.57 | 0.66 | -0.92 |  | 0.275 | 0.143 | 0.084 | 0.002 |
| SCP | 0.32 | -0.61 | -0.02 | -0.63 |  | 0.434 | 0.116 | 0.964 | 0.099 |
| CP | -0.85 | -0.13 | -0.78 | 0.61 |  | 0.011 | 0.761 | 0.029 | 0.116 |
| PTR | -0.44 | 0.32 | 0.78 |  |  | 0.275 | 0.434 | 0.029 |  |
| SS | -0.71 | -0.12 | 0.11 |  |  | 0.058 | 0.781 | 0.808 |  |
| DN | -0.71 | -0.18 | 0.18 |  |  | 0.058 | 0.673 | 0.673 |  |
| RN | -0.37 | -0.25 | 0.86 |  |  | 0.365 | 0.549 | 0.008 |  |
| SN | -0.80 | -0.49 | -0.37 |  |  | 0.021 | 0.222 | 0.365 |  |

Abbreviations:

All Spearman correlation coefficients $\rho$ and p-values for Figure 5 are provided when applicable for all investigated brain structures, middle cerebellar peduncle (MCP), pontine crossing tract (PCT), corticospinal tract (CS), medial lemniscus (ML), inferior cerebellar peduncle (ICP), superior cerebellar peduncle (SCP), cerebral peduncle (CP), posterior thalamic radiation (including optic radiation) (PTR), sagittal stratum (including inferior longitudinal fasciculus and inferior fronto-occipital fasciculus) (SS), and manually drawn volumes of interest for substantia nigra (SN), red nuclei (RN) and dentate nuclei (DN) and all contrasts, susceptibility mapping (QSM), $R_{1}$and $R_{2}$relaxometry, and fractional anisotropy (FA). Statistically significant findings are marked green.
