# Supplementary material for: Toward quantitative neuroimaging biomarkers for Friedreich's ataxia at 7 Tesla: Susceptibility mapping, diffusion imaging, R 2 and R 1 relaxometry
Source: J Neurosci Res. 2020 Jul 30;98(11):2219–31. doi: 10.1002/jnr.24701 (PMC7590084; doi:10.1002/jnr.24701)
Supplement: Supplementary file 2 — Transparent Peer Review Report [file JNR-98-2219-s002.docx]

**Towards quantitative neuroimaging biomarkers for Friedreich's ataxia at 7 Tesla: susceptibility mapping, diffusion imaging, R2 and R1 relaxometry**

Mangesius, Stephanie; Straub, Sina; Emmerich, Julian; Indelicato, Elisabetta; Nachbauer, Wolfgang; Degenhardt, Katja S.; Ladd, Mark; Boesch, Sylvia; Gizewski, Elke _____________________________________________________________________________________

| Review timeline: | Submission date: 29-Jan-2020  Editorial Decision: Major revision; 26-Mar-2020  Revision Received: 24-Feb-2020  Editorial Decision: Minor revision; 27-May-2020  Revision Received: 12-Jun-2020  Accepted: 8-July-2020 |  |
| --- | --- | --- |
|  |  |  |

Editor: Dr Jeremy Hogeveen

Reviewer 1: Rauscher, Alexander

Reviewer 2: Martinuzzi, Andrea

_____________________________________________________________________________________

1st Editorial Decision	26-Mar-2020

_____________________________________________________________________________________

26-Mar-2020

Dear Dr. Mangesius,

Thank you for submitting your work to the Journal of Neuroscience Research. 

The manuscript has now been reviewed by myself and two external expert reviewers. While the reviewers agree that this work represents a potential advancement, they have raised significant practical and conceptual concerns. 

Summarizing the comments, the reviewers were concerned with i: a lack of clarity regarding the potential benefit of 7T imaging for Friedreich Ataxia relative to standard 3T MRI, ii: justification of the multiple comparisons corrections approach, and iii: a better integration of these data with the prior literature.

Overall, in light of these concerns I am unable to accept the manuscript in its present form. That said, if you are able to address the reviewers' significant concerns, I would invite a heavily revised version of this work.

If you feel that you can adequately address the concerns of the reviewers, you may revise and resubmit your paper within 60 days. It will require further review. Please explain in your cover letter how you have changed the present version. If you require longer than 60 days to make the revisions, please contact Dr Cristina Ghiani (cghiani@mednet.ucla.edu). You can submit your revised manuscript directly by clicking on the following link: *** PLEASE NOTE: This is a two-step process. After clicking on the link, you will be directed to a webpage to confirm. *** 

<https://mc.manuscriptcentral.com/jnr?URL_MASK=ede286e2dde54624a5b420af916327c9>

Thank you again for your submission to the Journal of Neuroscience Research; we look forward to reading your revised manuscript.

Best Wishes, 

Dr Jeremy Hogeveen 
Associate Editor, Journal of Neuroscience Research

Dr Cristina Ghiani
Co-Editor-in-Chief, Journal of Neuroscience Research

____________________________________________________________________________________

Authors’ Response	24-Feb-2020

_____________________________________________________________________________________

PLACE THE REVIEWER COMMENTS AND RESPONSE TO REVIEWERS HERE

Dear Editors, dear reviewers,

Thank you very much for your detailed comments. We hope, that we could address all your concerns in sufficient detail in the revised manuscript. Additionally to addressing your comments, we included effect size of the group differences between healthy controls and patients, as obviously the low number of included subjects is the biggest limitation of this study. In the annotated manuscript changes are marked with reviewers’ comments numbers (R1.1 for the first comment of Reviewer 1, for example).

Reviewer: 1

Comments to the Author

In a constructive and professional manner, reviews should briefly describe the main claims and conceptual
advance, explain the strengths and weaknesses of the study and discuss areas in which there need to be
improvements in the clarity of communication.

The authors present a quantitative MRI study in Friedreich ataxia at 7T. R1, R2 relaxometry, diffusion, and
quantitative susceptibility mapping were performed in ten patients and ten controls. Significant differences
between patients and controls were found in 8/12 brain structures. In addition, voxel based morphometry
showed significant white matter atrophy in regions in the brain stem and cerebellum. Overall, this is a solid
paper in small cohort of patient with Friedreich's ataxia. However, given the significant findings across several
brain regions and rarity of the disease, 10 subjects seem justified.

1.1 If the authors are planning on recruiting more patients, I suggest to refer to the results as preliminary in the paper.

As FRDA is a rare disease, and considering the effort to transfer the patients from Austria to Germany for the measurements and the fact that not all patients who qualify for a clinical MRI, can safely be measured at 7 T, it might be a quite long process to recruit more patients and for this particular study, we do not intend to publish data of more patients. Therefore, we also provide effect size for group differences in the revised manuscript and emphasize that a larger study might be needed to find more structures for which the used methods could be biomarkers for pathological changes within these structures.

1.2 It seems that no corrections for multiple MRI methods and multiple brain regions were performed and p = 0.05 was used as threshold for significance. This is valid but it should be stated in the paper that the results generate hypotheses for these brain regions that would have to be confirmed in separate studies in different cohorts. I suggest weakening the statement related to statistically significant results at the beginning of the discussion.

Thank you for this comments. For Bonferroni correction, the p-value should be 0.005, however, the Bonferroni correction is very conservative. We change p for significance to 0.01 and mentioned this in the discussion.

1.3 The discussion should be expanded with respect to the pathophysiological meanings of the findings. In the introduction R2 is mentioned as being sensitive to myelin and iron but in the results section on page 8 the significant R2 are confined to structures with very high iron content and low myelin content. This absence of myelin related findings should be discussed in more detail.

Thank you for this comments, we added these aspects to the discussion.

1.4 I would like to see a short summary of the dictionary based T2 mapping approach.

A short summary was included.

1.5 Discussion and other sections of the paper: The term gender is used where it appears
    that sex was meant.

Gender was replaced by sex.

1.6 I am not sure I understand the discussion about age related changes in QSM. The controls are perfectly matched to the patients and age related changes should be present in both groups but linear regressions for chi(age) may differ between the groups.

We tried to clarify this point in the discussion. Now it should be more comprehensive that within the patient group, when comparing susceptibility values for e.g. DN, we would expect higher values for severer disease/high number of GAA repeats/early onset. But at the same time, we would expect higher values for older patients. However, most of them had a much later age of onset/ shorter GAA repeats (which in other studies correlated with disease severity). Therefore, they likely suffer from a less severe disease. Consequently, higher values may indicate increased disease severity or older patients. Further studies including patients at the same age with different GAA triplet repeats (e.g. different disease severity) are needed to clarify this aspect.

1.7 Figures: I like the idea of showing these matrices for correlations between quantitative MRI and disease characteristics. However, I would like to see a table as well, at least as supplementary material.

We included a Table as supplementary material.

1.8 The color bars are labeled with rho but not with p. (e.g. in the fig on p31).

We changed this, thank you.

Reviewer: 2

Comments to the Author

The authors present a novel 7T multimodal imaging study of a small group of rater advanced FA patients. The study has the obvious interest of the high field approach to a rare disease, but has substantial weaknesses that prevent its acceptance in the present form.

2.1 The main problem the reader faces when going through the paper is the relative confusion in data presentation and discussion, which leaves the reader asking in the end what is the real value of the 7T imaging over the traditional 3T scanning. This is particularly relevant when considering some well-established and confirmed elements (such as the Dentate nucleus involvement) which doesn't appear to be confirmed in the present study. The interpretation of the data should also keep into consideration the demographics and clinical stage of the participants, especially considering the paucity of numbers. It has been shown recently (Salvadurai et al 2019) that the various MRI indices likely represent different pathology processes differently intercepted in the various patients according to age, disease stage and severity, brain area. It would be thus appropriate the authors first of all update the references (works like Vavla et al 2018, Salvadurai et al 2018, 2019 and 2020 are not considered) and present more clearly the background of current knowledge on MRI phenotype in FA, secondly match their data with the existing body of evidence (both in-vivo and pathology based), finally present the new elements stemming from their study and discuss them offering alternative possible explanations/interpretations.

Thank you very much for this comment, we added the literature and discussed our results in more detail with respect to this literature and the disease characteristics in the patients including age at onset and GAA repeats. Since Reviewer 1 has not been sure about the relevance of age related changes in QSM as the controls are perfectly matched to the patients, we hope we did right by both reviewers by explaining this aspect as suggested by Reviewer 1 and adding literature as suggested by Reviewer 2.

2.2 MP2RAGE is a T1-weighted image, while the reference image for the JHU atlas included in FSL is a T2-weighted one (Note that the JHU atlas included in FSL is not in the MNI space, as it can be seen comparing the corpus callosum in the JHU atlas and in the MNI images).

We apologize for the confusion, we used the JHU-ICBM-DTI-81 white-matter labels atlas which is titles with ‘JHU DTI-based white-matter atlases’ (not the JHU white-matter tractography atlas). This is a standard-space average. We clarified this in the manuscript. We are aware that although the ICBM-DTI-81 template is in standard space, it’s not a standard space template, however, as we performed a VOI-based analysis the labels of this template (that did not differentiate between hemispheres), errors can be expected to be minimal. We used the MP2RAGE data and registered the MNI template to it, because the MP2RAGE data, was the data with the largest brain coverage. Previously, we tried to register the FA maps to the JHU FA atlas data and then subsequently to the other contrasts which generated unusable results due to insufficient accuracy in registration (both for linear and non-linear registration). We think this is beyond the scope of this manuscript and decided not to include this in the study. The accurate placement of the labels was visually inspected for all contrasts.

2.3 The application of a non-linear transformation (FNIRT) between images with different contrasts can be a source of errors, most of all when focusing on small structures, not easily detectable in the structural images. Since the authors acquire also structural T2W images, I would suggest to perform the non-linear registration between a couple of T2W images.

In a preliminary step of this study, registration was both performed with FLIRT and FNIRT, and registration accuracy was visually assessed for all subjects and contrasts. The FNIRT registration was favorable and therefore chosen. We include this in the revised manuscript. We only used FNIRT for registering images with the same contrast (MP2RAGE to MNI). Using T2w or DTI images was unfeasible, because of the anisotropic resolution (slice thickness) and limited brain coverage, this was assessed preliminary to this study.

2.4 What rules were applied to define the VOI? What threshold were applied? Were they common among subjects or subject based?

We explained the rules that we applied in detail in the revised manuscript. They were common for all patients/volunteers.

2.5 The acquisition of a Diffusion Weighted sequence and its preprocessing is completely absent in the material and methods section.

Thank you for your comment, we added details to make the manuscript for self-contained.

2.6 Why did authors decide to use the Wilcox ranksum test? Non-parametric tests have a smaller statistical power than parametric test, most of all in small datasets, like in this study.

Thank you, we changed this, and use ANCOVA in the revised manuscript with age and sex as covariates. Only for the covariate age, R1, and substantia nigra significance was found. We included this in the manuscript.

2.7 Did authors perform any multiple comparison correction in the VOI based statistical analysis?

For the VOI-based analysis no multiple comparisons correction was used, however, in the revised manuscript, we changes p to 0.01 instead of 0.05 and explained this in the discussion.

2.8 Why did the authors included gender and age as covariates only for the VBM analysis and not also in the VOI based analysis?

Thank you for your comment, we use ANCOV with age and gender as covariates for the VOI-based analysis as well in the revised manuscript.

2.9 Why the FA was not computed for all VOI?

Due to SAR and acquisition time constraints, the brain coverage (field of view in z-direction) of the DTI sequence was smaller than, for example, for the MP2RAGE sequence, therefore not all VOIs could be covered in all volunteers. These VOIs were excluded for DTI.

2.10 P-value colorbar should be set to better differentiate small p-values. P-values very high, larger than 0.1, are not informative and the colorbar should focus on smaller ones.

Thank you for your comment, we adapted the colorbars.

2.11 Caption should specify that white marks show significant p-values.

Thank you, we included this.

2.12 Size of the shown SCP looks quite small: the slice chosen to show SCP seems maybe too high

In Figure 1 and 4, slices were chosen such that as many structures as possible could be shown with a small number of images. The appearance of SCP could also vary with slice orientation, in this study slices were aligned strictly transversal.

2^nd^ Editorial Decision	27-May-2020

_____________________________________________________________________________________

Dear Dr. Mangesius,

Thank you for sending us your revision. The revised manuscript has now been reviewed by myself and both original reviewers. While all reviewers agree that you have done an excellent job addressing most of the concerns raised during the first round of review, there are several remaining concerns that need to be addressed before the manuscript will be acceptable for publication in the Journal of Neuroscience Research.

In particular,

i: further elaboration on the DTI analysis pipeline must be provided, and

ii: please elaborate on the importance of 7T imaging biomarkers to the field in the intro and discussion (both with respect to Friedrich's ataxia, as well as other neurological disorders; i.e. WHY are 7T biomarkers useful and what diagnostic validity might they provide beyond 3T?).

If you are able to address the reviewers' remaining concerns, I would invite a revised version of the manuscript.

I expect that these points should be relatively straightforward to address, but if there are any questions or points that are problematic, please feel free to contact me. I am glad to discuss.

We ask that you return your manuscript within 30 days. Please explain in your cover letter how you have changed the present version. If you require longer than 30 days to make the revisions, please contact Dr Cristina Ghiani (cghiani@mednet.ucla.edu). You can submit your revised manuscript directly by clicking on the following link: *** PLEASE NOTE: This is a two-step process. After clicking on the link, you will be directed to a webpage to confirm. ***

<https://mc.manuscriptcentral.com/jnr?URL_MASK=ef808e3eef0f45debc3aca9b0e48cab8>

Thank you again for your submission to the Journal of Neuroscience Research; we look forward to reading your revised manuscript.

Best Wishes,

Dr Jeremy Hogeveen

Associate Editor, Journal of Neuroscience Research

Dr Cristina Ghiani

Co-Editor-in-Chief, Journal of Neuroscience Research Associate Editor: Hogeveen, Jeremy Comments to the Author:

Dear Dr. Mangesius,

Thank you for sending us your revision. The revised manuscript has now been reviewed by myself and both original reviewers. While all reviewers agree that you have done an excellent job addressing most of the concerns raised during the first round of review, there are several remaining concerns that need to be addressed before the manuscript will be acceptable for publication in the Journal of Neuroscience Research. In particular, i: further elaboration on the DTI analysis pipeline must be provided, and ii: please elaborate on the importance of 7T imaging biomarkers to the field in the intro and discussion (both with respect to Friedrich's ataxia, as well as other neurological disorders; i.e. WHY are 7T biomarkers useful and what diagnostic validity might they provide beyond 3T?).

If you are able to address the reviewers' remaining concerns, I would invite a revised version of the manuscript.

Best Wishes,

Dr Jeremy Hogeveen

Associate Editor, Journal of Neuroscience Research

_____________________________________________________________________________________

Authors’ Response

8-Jun-2020

_____________________________________________________________________________________

Dear Editors, dear reviewers,

Thank you very much for your detailed comments.

The manuscript has now i: further elaborated on the DTI analysis pipeline, and ii: discussed the importance of 7 T imaging biomarkers to the field with respect to Friedrich's ataxia, as well as other neurological disorders, especially why 7 T biomarkers are useful and what diagnostic validity they might they provide beyond 3 T imaging. We have considered all additional comments and modified the manuscript accordingly.

Editor:

E.1	further elaboration on the DTI analysis pipeline must be provided, and

Thank you, for this comment, we have added the necessary details in the Methods section and the Discussion.

E.2	please elaborate on the importance of 7T imaging biomarkers to the field in the intro and discussion (both with respect to Friedrich's ataxia, as well as other neurological disorders; i.e. WHY are 7T biomarkers useful and what diagnostic validity might they provide beyond 3T?).

We have furthermore elaborated on the importance of 7 T MRI in finding appropriate biomarkers both in the Introduction and the Discussion.

Reviewer: 1

Comments to the Author

I have no further comments.

Reviewer: 2

Comments to the Author

The authors have satifacted most of the reviewers suggestions. However, still some points should be clarified:

2.1	DTI on a 7T magnet is quite complicate and not so reliable due to the high rate of artifacts. The authors mention a couple of sequence parameters but they do not provide details of the validation protocol (is that taken from the literature or authonomously elaborated?) The authors state that the FA values have been obtained using the vendor's software. These softwares are inapporpriate for extracting data with the quality requested for research due to insufficient image correction. This major limitation should be mentioned in the text coutioning the readers on the solidity of the FA data presented.

Thank you for noticing this methodological aspect, this comment makes an important point, which we now addressed accordingly. Due to severe artifacts that are present when using standard single-shot EPI sequences at ultra-high field, especially in regions such as the brainstem, a segmented EPI sequence with navigator-based reacquisition was used, all sequence parameters are provided in the revised manuscript as well as citations for the sequence. We have now stressed this in the discussion. Moreover, to again check the integrity of our data, we performed a DTI analysis in FSL using eddy and compared the resulting FA maps with the ones that we were using in this study. Please, see the figures below, which show in the first row, the FSL-corrected data, in the second row, the vendor-provided data and in the third row the difference. We randomly chose two slices (Figure 1 and Figure 2), and five patients/ controls (columns).

Figure 1:

Figure 2:

In conclusion, we could not observe considerable differences between the two methods, DTI analysis in FSL using eddy and compared the resulting FA maps (as suggested by the reviewer) with the ones that we were using in this study. We acknowledge this important methodological point, and discussed this aspect accordingly in the revised manuscript.

2.2	Finally, the significance and added value of the 7T study over the usual 3T should be discussed and presented. Still the reader is left wondering why going through the trouble and cost of doing a 7T study if no major relevant new elements can be extracted from it.

We are grateful to the referee for raising this point and comment on this issue in the revised version of our discussion of limitations.
